# Supplementary material for: Changes in care-seeking for common childhood illnesses in the context of Integrated Community Case Management (iCCM) program implementation in Benishangul Gumuz region of Ethiopia
Source: PLoS One. 2020 Nov 13;15(11):e0242451. doi: 10.1371/journal.pone.0242451 (PMC7665800; doi:10.1371/journal.pone.0242451)
Supplement: S1 Table — (DOCX) [file pone.0242451.s001.docx]

**
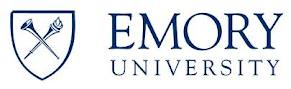
** **Emory University, ICCM and CBNC Benishangul Gumuz Project**

**ICCM Household Survey Questionnaire V 1.1**

| **Introduction and Consent Form** | | | |
| --- | --- | --- | --- |
| I [name of worker] ______________________ am working for Emory University, iCCM/CBNC project. We are doing a house to house survey on integrated community case management (iCCM). You can take part in the survey as you have a child whose age is less than five years old. If you are interested to participate in the study, I would like to ask you and learn about child health and the care practices provided for a child. The in-depth interview may take about 30 minutes. You may not benefit from participating in this interview as an individual. However, your participation and the information you provide will help us understand issues about child health care and improve the child health service delivery in your community. Your participation in the study will not have any risk or problem. Whether you participate in this study, it also doesn’t influence the health service you will get. The information that you provide us will be kept private and confidential; name and other Information identifying you are not part of the questionnaire and not recorded. Your participation is entirely voluntary. You do not have to take part in this interview if you do not wish to do so, and you may also stop participating in the interview or may not answer a particular question that you are not comfortable.  If you have any concerns or question about the sudy, please let me know and I will explain you.  Do you get informed consent obtained:  **1 = Yes 2 = No**  __________________________________ _________________ _________________  Full name of person who take permission Signature Date | | | |
| **Section I: GENERAL INFORMATION ON INTERVIEW** | | | |
| **S.N** | **Questions** | **Response** |  |
| **100** | Questionnaire number (include Woreda, Kebele and HH code) | Questionnaire # \|_____\|_____\|_____\| |  |
| **101** | Woreda Name | 01 = Asossa  02 = Banbasi  03 = Homosha  04 = Kurmuk  05 = Menge  06 = Sherkole  07 = Odda |  |
| **102** | Kebele Name | _________________ |  |
| **103** | Gote name | _________________ |  |
| **104** | Interviewer full name | _________________ |  |
| **105** | Date Interview Performed *(DD/MM/YY)* | _________________ |  |
| **106** | Time Interview Performed *(hh/mm)* | _________________ |  |
| **107** | Number of HH members available during the interview | 01 = one  02 = two  03 = three and above |  |
| **108** | Relationship of respondent with the child | 01 = Mother  02 = Other (specify) ________________ |  |
| **109** | Child first name | ___________________ |  |

| **S.N** | **Questions** | | | **Response** | | | | | | | | | | |  |
| --- | --- | --- | --- | --- | --- | --- | --- | --- | --- | --- | --- | --- | --- | --- | --- |
| **Section II: BACKGROUND AND HOUSEHOLD CHARACHTERISTICS** | | | | | | | | | | | | | | | |
| **Interview Say: “I am going to start by asking you some questions about you and your household.”** | | | | | | | | | | | | | | | |
|  | How old are you?  (age in completed years) | | | [______]years  99 = Don’t Know | | | | | | | | | | |  |
|  | How long have you been living continuously in (NAME OF CURRENT PLACE OF RESIDENCE)?  *IF LESS THAN ONE YEAR, RECORD '00' YEARS.* | | | [_______] years [______] months  88 = Other(Specify)_______________ | | | | | | | | | | |  |
|  | What is your religion? | | | 01 = Muslim  02 = Christian orthodox  03 = Christian protestant  04 = Christian (other)  05 = Traditional  88 = Other(Specify)_______________ | | | | | | | | | | |  |
|  | What is your ethnicity? | | | 01 = Berta  02 = Gumuz  03 = Shinasha  04 = Amhara  05 = Oromo  06 = Tigre  88 = Other(Specify)_______________ | | | | | | | | | | |  |
|  | What is your current marital status? | | | 01 = Married  02 = Live with cohabit  03 = Separated/Divorced  04 = Widowed  05 = Single | | | | | | | | | | |  |
|  | Are you able to read and write? | | | 01 = Yes  02 = No | | | | | | | | | | | **2🡪208** |
|  | Did you ever attend formal school? | | | 01 = Yes  02 = No | | | | | | | | | | | **2🡪208** |
|  | Educational status- what is the highest grade you completed? | | | 01 = Grade [___\|___]  13 = Technical/vocational certificate  14 = University/college diploma  15 = University/college Degree or Higher | | | | | | | | | | |  |
|  | How many people reside in this household? | | | [_____] | | | | | | | | | | |  |
|  | Did you earn any cash income during last month? | | | 01 = Yes  02 = No | | | | | | | | | | |  |
|  | Did your spouse or husband earn any cash income during the last month? | | | 01 = Yes  02 = No  98 = Don’t know  99 = Not Applicable | | | | | | | | | | |  |
|  | What is the total amount of income your families earned during last month?  *If No income during last month please insert “00”* | | | [______________] | | | | | | | | | | |  |
|  | From which main material is the household floor made?  **Record observation.** | | | 01 = Natural floor (earth/sand/dung)  02 = Rudimentary floor (wood/palm/bamboo)  03 = Finished floor (polished wood/ /tiles/cement/carpet)  88 = Other (Specify)___________________ | | | | | | | | | | |  |
|  | From which main material is the household roof made?  **Record observation** | | | 01 = Thatch/grass or leaves  02 = Plastic sheet  03 = completed roof (Sheet metal, tiles…)  88 = Other (Specify)___________________ | | | | | | | | | | |  |
|  | From which main material is the household walls made?  **Record Observation** | | | 01 = No walls  02 = Natural materials (cane, wood, mud, straw)  03 = Stone with mud  04 = Complete wall (Stone/bricks with cement…)  88 = Other (Specify)___________________ | | | | | | | | | | |  |
|  | How many rooms in this HH are used for sleeping? | | | [ _____________ ] rooms | | | | | | | | | | |  |
|  | What is the main source of drinking water for members of your household? | | | Piped water  01 = Piped into dwelling  02 = Piped into compound  03 = Piped outside compound  Well water  04 = Protected Well  05 = Unprotected Well  06 = Open Well  Spring water  07 = Protected Spring  08 = unprotected Spring  Surface water  09 = River  10 = Pond/Lake/Dam  11 = Rainwater | | | | | | | | | | |  |
|  | How long does it take you to get drinking water and bring it home? | | | Minutes [____\|____]  Hours [____\|____]  96 = On premises | | | | | | | | | | |  |
|  | Do you treat your water in any way to make it safer to drink? | | | 01 = Yes  02 = No | | | | | | | | | | | **2 🡪220** |
|  | What do you mostly do to make water safe to drink? | | | 01 = Boiling  02 = adding chemicals like Wuha-agar  03 = Use water filter *(ceramic, sand composite*  04 = Strain it through a clot  05 = Using gravel and sand  06 = Solar disinfection  07 = Let it stand and settle  88 = Other (specify)________________  99 = Do not Know | | | | | | | | | | |  |
|  | What kind of toilet facility do most of your family members use? | | | 01 = Pit Latrine/traditional pit toilet  02 = Ventilated improved pit latrine (VIP)  03 = Flush toilet  04 = No facility/Bush/Field  88 = Other(Specify)____________________ | | | | | | | | | | |  |
|  | Where did you last defecate/ passed stool? | | | 1 = Pit Latrine/traditional pit toilet  2 = Ventilated improved pit latrine (VIP)  3 = Flush toilet  4 = No facility/Bush/Field  5 = Other(Specify)____________________ | | | | | | | | | | |  |
|  | Which utilities do your family own? | | | **Variables** | | | | | | **Yes** | | **No** | | |  |
|  |  |  |  | Electricity? | | | | | | 01 | | 02 | | |  |
|  |  |  |  | Solar power? | | | | | | 01 | | 02 | | |  |
|  |  |  |  | Wrist watch? | | | | | | 01 | | 02 | | |  |
|  |  |  |  | Radio? | | | | | | 01 | | 02 | | |  |
|  |  |  |  | Television? | | | | | | 01 | | 02 | | |  |
|  |  |  |  | Mobile telephone? | | | | | | 01 | | 02 | | |  |
|  |  |  |  | Landline telephone? | | | | | | 01 | | 02 | | |  |
|  |  |  |  | Refrigerator? | | | | | | 01 | | 02 | | |  |
|  |  |  |  | Table? | | | | | | 01 | | 02 | | |  |
|  |  |  |  | Chair? | | | | | | 01 | | 02 | | |  |
|  |  |  |  | Bed with cotton/sponge mattress? | | | | | | 01 | | 02 | | |  |
|  |  |  |  | Electric Stove? | | | | | | 01 | | 02 | | |  |
|  |  |  |  | Kerosene Stove or gas stove? | | | | | | 01 | | 02 | | |  |
|  |  |  |  | Kerosene lamp/pressure lamp? | | | | | | 01 | | 02 | | |  |
|  |  |  |  | Kitchen? | | | | | | 01 | | 02 | | |  |
|  |  |  |  | Bicycle? | | | | | | 01 | | 02 | | |  |
|  |  |  |  | Motorcycle or motor scooter? | | | | | | 01 | | 02 | | |  |
|  |  |  |  | Animal-drawn cart? | | | | | | 01 | | 02 | | |  |
|  |  |  |  | Car or truck? | | | | | | 01 | | 02 | | |  |
|  | Does this household own any livestock, herds, or farm animals? | | | 01 = Yes  02 = No | | | | | | | | | | | **02🡪225** |
|  | How many of the following animals, does this household own?  *(If none record “00”)* | | | [_______]cows, oxen  [_______] Camels  [_______] Horses  [_______] Donkeys  [_______] Mules  [_______] Goats  [_______] Sheep  [_______] Chicken  [_______] Other _________________ | | | | | | | | | | |  |
|  | Does this household/Family own LOCAL UNITS of agricultural land? | | | 1 = Yes  2 = No | | | | | | | | | | | **02🡪300** |
|  | **If yes, Q. 225,** how many LOCAL UNITS of agricultural land do you own?  *If unknown, record “99”* | | | ________ amount __________ local Units  89 = Not comfortable  99 = Don’t know | | | | | | | | | | |  |
| **Section III: BIRTH HISTORY** | | | | | | | | | | | | | | | |
| **Interview Say***: “Now I would like to ask you some questions about your experiences with pregnancy and childbirth.”* | | | | | | | | | | | | | | | |
|  | | | How many times were you pregnant? | | [_____] times  99 = Don’t Know | | | | | | | | |  | |
|  | | | How many live births you have given during your life time? | | [_____] times  99 = Don’t Know | | | | | | | | |  | |
|  | | | How many sons and daughters do you have living with the family now? | | [_____] sons < 5 years AND  [_____] daughters <5 years  [_____] total number of < 5 years | | | | | | | | |  | |
|  | | | Have you ever had a pregnancy that didn’t end up in a live birth? | | 01 = Yes  02 = No | | | | | | | | | **2 🡪306** | |
|  | | | If yes **Q. 303**, how many pregnancies have you face that didn’t end up in live birth? | | [_____] pregnancies | | | | | | | | |  | |
|  | | | How many pregnancies you lost before they were ***7 months (28 weeks)?*** | | [_____] pregnancies | | | | | | | | |  | |
|  | | | Have you ever had a child who died before the ***age of one year***? | | 01 = Yes  02 = No | | | | | | | | | **2 🡪310** | |
|  | | | Have you ever had a child who died in the ***first 28 days*** of life? | | 01 = Yes  02 = No | | | | | | | | | **2 🡪310** | |
|  | | | Have you ever had any child who died in the ***first seven days*** of his/her life? | | 01 = Yes  02 = No | | | | | | | | | **2 🡪310** | |
|  | | | Were any of the children who died in the ***first 48 hours*** of his/her life? | | 01 = Yes  02 = No | | | | | | | | |  | |
|  | | | Are you currently pregnant? | | 01 = Yes  02 = No | | | | | | | | | **1 🡪314** | |
|  | | | Have you currently planned to become pregnant? | | 01 = Yes  02 = No | | | | | | | | |  | |
|  | | | If no for **Q. 310**, are you currently using a family planning method to avoid or delay pregnancy? | | 01 = Yes  02 = No | | | | | | | | | **2 🡪400** | |
|  | | | Which of the following family planning method are you using currently? | | 01 = Oral contraceptive Pills  02 = Condom (male)  03 = Condom (female)  04 = Male sterilization  05 = Female sterilization  06 = IUD  07 = Rhythm method  08 = Abstinence from sex  09 = Implants  10 = Injectable (DEPO)  11 = Diaphragm  12 = Withdrawal method  13 = Lactational amenorrhea /Breast feeding  14 = Others (Specify) __________________ | | | | | | | | |  | |
|  | | | Do you know where you can get a family planning method? | | 01 = Yes  02 = No | | | | | | | | | **2 🡪400** | |
|  | | | Where do you think family planning methods could be obtained?  (You can give multiple answers above one, Read the choices and ask further by saying “what else”) | | 01 = Health Post  02 = Health Center  03 = Government Hospital  04 = Non-Governmental clinic  05 = Private Hospital  06 = Private clinic  07 = Private pharmacy  08 = Community based outlets  09 = Rural drug vendor  10 = Traditional practitioner  11 = Holy water/holy place  12 = Shop  13 = Other (specify) ____________________ | | | | | | | | |  | |
| **Section IV: Awareness about health facilities and utilization of health services** | | | | | | | | | | | | | | | |
| **Interviewer Say***: “Now I would like to ask you some questions about your trust of the health care providers and community volunteers in the facilities you utilize.”* | | | | | | | | | | | | | | | |
|  | | Does the kebele have health facility? | | 01 = Yes  02 = No  99 = Don’t know | | | | | | | | | | |  |
|  | | What type of facility is this (the nearest)? | | 01 = Health Post  02 = Health Center  03 = Government Hospital  04 = Non-Governmental clinic  05 = Private Hospital  06 = Private clinic  88 = Other (specify) ____________________  99 = Don’t Know | | | | | | | | | | |  |
|  | | How long does it take you to walk to the nearest health facility?  *[If less than an hour record it in Minutes, if you are not Shure record “99” Hr.]* | | Health post: Minutes [__\|___] Hours [__\|__] | | | | | | | | | | |  |
|  |  |  |  | Health Center: Minutes [__\|_ _] Hours [__\|__] | | | | | | | | | | |  |
|  | | Does your kebele have health post/health center? | | 01 = Yes  02 = No  99 = Don’t know | | | | | | | | | | |  |
|  | | Have you ever visited the health post/health facility at any time in the last one year? | | 1 = Yes  2 = No  8 = No health facility access | | | | | | | | | | | **2🡪407**  **8🡪407** |
|  | | When was the last time you visited the health facility post? | | 01 = Within last 1 month  02 = With last 2-3 months  03 = Within 4-6 months  04 = > 6 months ago | | | | | | | | | | |  |
|  | | What was the main reason for you to visit the health facility post lastly?  **If mentioned (Yes); If not mentioned (No)**  **Do not read the responses**  **(Multiple Responses Possible)** | | **Reasons** | | | | | | **Yes** | | | **No** | |  |
|  |  |  |  | Family planning | | | | | | 01 | | | 02 | |  |
|  |  |  |  | ANC follow up | | | | | | 01 | | | 02 | |  |
|  |  |  |  | Delivery Service | | | | | | 01 | | | 02 | |  |
|  |  |  |  | PNC service | | | | | | 01 | | | 02 | |  |
|  |  |  |  | Child immunization | | | | | | 01 | | | 02 | |  |
|  |  |  |  | Growth monitoring | | | | | | 01 | | | 02 | |  |
|  |  |  |  | treatment of my sick child | | | | | | 01 | | | 02 | |  |
|  |  |  |  | diarrhea treatment | | | | | | 01 | | | 02 | |  |
|  |  |  |  | fever/malaria treatment | | | | | | 01 | | | 02 | |  |
|  |  |  |  | Pneumonia treatment | | | | | | 01 | | | 02 | |  |
|  |  |  |  | Treatment of a neonate | | | | | | 01 | | | 02 | |  |
|  |  |  |  | Receive bed nets | | | | | | 01 | | | 02 | |  |
|  |  |  |  | For health education | | | | | | 01 | | | 02 | |  |
|  |  |  |  | Other (Specify) ______________ | | | | | |  | | |  | |  |
|  | | Have you heard about the health extension workers? What about nurses? | | 01 = Yes  02 = No | | | | | | | | | | | **02🡪411** |
|  | | Have they visited your house? | | 01 = Yes  02 = No  99 = Don’t know or Not sure | | | | | | | | | | | **2 🡪410**  **99🡪 410** |
|  | | If **Q. 408 is “yes**”, when was the last time the HEW visited your house? | | 01 = Within last month  02 = Within last 2 – 3 months  03 = Greater than 3 months ago | | | | | | | | | | |  |
|  | | How much do you trust the ***HEW*** to provide care for your under-five child? | | 01 = I always trust them  02 = I sometimes trust them  03 = I don’t trust them | | | | | | | | | | |  |
|  | | Have you heard of or do you know about the Frontline Workers? | | 01 = Yes  02 = No | | | | | | | | | | | **2 🡪415** |
|  | | Have they **(FLWs)** visited your house over the past 6 months? | | 01 = Yes  02 = No | | | | | | | | | | | **2 🡪414** |
|  | | **If your answer to Q. 412 is “yes”**, When was your house last visited by FLW? | | 01 = Within last month  02 = Within last 2 – 3 months  03 = Greater than 3 months ago | | | | | | | | | | |  |
|  | | How much do you trust a ***FLW*** to provide care for your under-five child? | | 01 = I always trust them  02 = I sometimes trust them  03 = I don’t trust them | | | | | | | | | | |  |
|  | | How much do you trust a ***nurse*** at the health post to provide care for your under-five child? | | 01 = I always trust them  02 = I sometimes trust them  03 = I don’t trust them  08 = No nurses at the HP | | | | | | | | | | |  |
|  | | How much do you trust ***Voluntary Community Health workers*** to provide care for your under-five child? | | 01 = I always trust them  02 = I sometimes trust them  03 = I don’t trust them  08 = I don’t know the existence community volunteers | | | | | | | | | | |  |
|  | | In the last 6 months have you ever received service or advice from TBAs? | | 01 = Yes  02 = No | | | | | | | | | | | **2 🡪420** |
|  | | What advice or service did the TBA gave you when she last visited your house?    **If mentioned (Yes); If not mentioned (No)**  **Do not read the responses**  **(Multiple Responses Possible)** | | **Advice or information on** | | | | | | **Yes** | | | **No** | |  |
|  |  |  |  | Immunization | | | | | | 01 | | | 02 | |  |
|  |  |  |  | Child nutrition | | | | | | 01 | | | 02 | |  |
|  |  |  |  | Diarrhea treatment | | | | | | 01 | | | 02 | |  |
|  |  |  |  | Care seeking for sick child | | | | | | 01 | | | 02 | |  |
|  |  |  |  | Family planning | | | | | | 01 | | | 02 | |  |
|  |  |  |  | Antenatal care follow up | | | | | | 01 | | | 02 | |  |
|  |  |  |  | Skilled birth attendance | | | | | | 01 | | | 02 | |  |
|  |  |  |  | Postnatal care follow-up | | | | | | 01 | | | 02 | |  |
|  |  |  |  | HIV/AIDS | | | | | | 01 | | | 02 | |  |
|  |  |  |  | Hygiene | | | | | | 01 | | | 02 | |  |
|  |  |  |  | Promotion pit latrine construction | | | | | | 01 | | | 02 | |  |
|  |  |  |  | Promotion on latrine use | | | | | | 01 | | | 02 | |  |
|  |  |  |  | Promotion on safe water use | | | | | | 01 | | | 02 | |  |
|  |  |  |  | Did not receive advice or service | | | | | | 01 | | | 02 | |  |
|  |  |  |  | Don’t know | | | | | | 01 | | | 02 | |  |
|  |  |  |  | Other, specify________________ | | | | | | 01 | | | 02 | |  |
|  | | How much do you trust a ***Traditional Birth Attendants*** to provide care for your under-five child? | | 01 = I always trust them  02 = I sometimes trust them  03 = I don’t trust them | | | | | | | | | | |  |
|  | | Have you heard of or do you know about **Health Development Army /HDAs/** in your community? | | 01 = Yes  02 = No | | | | | | | | | | | **02 🡪425** |
|  | | Are you a member of **HDA** in your kebele? | | 01 = Yes  02 = No | | | | | | | | | | |  |
|  | | During the last six months did any member of HDA visit your home to talk about health-related issues? | | 01 = Yes  02 = No | | | | | | | | | | | **02 🡪425** |
|  | | When the HDA last visited your home, what information /advice did he/she give you? In which areas did he/she give you information/Advice?  **If mentioned (Yes); If not mentioned (No)**  **Do not read the responses**  **(Multiple Responses Possible)** | | **Advice or information on** | | | | | | **Yes** | | | **No** | |  |
|  |  |  |  | Immunization | | | | | | 01 | | | 02 | |  |
|  |  |  |  | Child nutrition | | | | | | 01 | | | 02 | |  |
|  |  |  |  | Diarrhea treatment | | | | | | 01 | | | 02 | |  |
|  |  |  |  | Care seeking for sick child | | | | | | 01 | | | 02 | |  |
|  |  |  |  | Family planning | | | | | | 01 | | | 02 | |  |
|  |  |  |  | Antenatal care follow up | | | | | | 01 | | | 02 | |  |
|  |  |  |  | Skilled birth attendance | | | | | | 01 | | | 02 | |  |
|  |  |  |  | Postnatal care follow-up | | | | | | 01 | | | 02 | |  |
|  |  |  |  | HIV/AIDS | | | | | | 01 | | | 02 | |  |
|  |  |  |  | Hygiene | | | | | | 01 | | | 02 | |  |
|  |  |  |  | Promotion pit latrine construction | | | | | | 01 | | | 02 | |  |
|  |  |  |  | Promotion on latrine use | | | | | | 01 | | | 02 | |  |
|  |  |  |  | Promotion on safe water use | | | | | | 01 | | | 02 | |  |
|  |  |  |  | Did not receive advice or service | | | | | | 01 | | | 02 | |  |
|  |  |  |  | Don’t know | | | | | | 01 | | | 02 | |  |
|  |  |  |  | Other, specify_________________ | | | | | | 01 | | | 02 | |  |
|  | | Have you heard of or do you know about social mobilization committee /SMC/ in your community? | | 01 = Yes  02 = No | | | | | | | | | | |  |
|  | | Are you a member of **SMC** in your kebele? | | 01 = Yes  02 = No | | | | | | | | | | |  |
|  | | During the last six months did any member of **SMC** visit your home to talk about health-related issues? | | 01 = Yes  02 = No | | | | | | | | | | |  |
|  | | Out of the danger signs of under-five children on which ones were you given an advice?  **(Multiple responses are possible,**  **Do not read the responses)** | | 01 = Unable to drink or breastfeed  02 = Convulsion  03 = Lethargic /unconscious  04 = Vomits everything  05 = Fever  06 = Hypothermia  07 = Sunken eye  08 = Skin pinch going back slowly  09 = Complicated measles  10 = Fast breathing/breathing difficulty  11 = Severe chest in-drawing  12 = Jaundice in 24 hours of birth  13 = Jaundice after 14 days  14 = Severe persistent diarrhea (>14 days)  15 = Dysentery  88 = Other (specify) ____________________  99 = Don’t Know | | | | | | | | | | |  |
| **Section V: Child Health and Care seeking behavior** | | | | | | | | | | | | | | | |
| **Interviewer:** *Now I want to talk to you about your child health and care seeking behavior [Child name]*  please use the baby name or baby ID | | | | | | | | | | | | | | | |
|  | | Age of the child in month? (in complete months)  *If less than a month write 00* | | [___\|___] months | | | | | | | | | | |  |
|  | | Sex of the child | | 01 = Male  02 = Female | | | | | | | | | | |  |
|  | | Have you ever breastfeed (NAME)? | | 01 = Yes  02 = No | | | | | | | | | | | **2🡪505** |
|  | | Are you still breastfeeding? | | 01 = Yes  02 = No | | | | | | | | | | |  |
|  | | For how many months did you(NAME) breastfeed? | | [_______] months  99 = I don’t know | | | | | | | | | | |  |
|  | | Did you ever have immunization card for child (NAME)? | | 01 = Yes  02 = No  08 = Not sure | | | | | | | | | | | **2🡪508** |
|  | | Do you have a card where the immunization status of NAME) is registered? | | 01 = Yes  02 = No | | | | | | | | | | | **2🡪508** |
|  | | Register/list all the immunization date for each vaccine from the immunization card. | | **Vaccine** | | | **Yes** | | **No** | **Date** | | | | |  |
|  |  |  |  | BCG | | | 01 | | 02 | \|____\|____\|____\| | | | | |  |
|  |  |  |  | POLIO 0 | | | 01 | | 02 | \|____\|____\|____\| | | | | |  |
|  |  |  |  | POLIO 1 | | | 01 | | 02 | \|____\|____\|____\| | | | | |  |
|  |  |  |  | POLIO 2 | | | 01 | | 02 | \|____\|____\|____\| | | | | |  |
|  |  |  |  | POLIO 3 | | | 01 | | 02 | \|____\|____\|____\| | | | | |  |
|  |  |  |  | PENTA1 | | | 01 | | 02 | \|____\|____\|____\| | | | | |  |
|  |  |  |  | PENTA2 | | | 01 | | 02 | \|____\|____\|____\| | | | | |  |
|  |  |  |  | PENTA3 | | | 01 | | 02 | \|____\|____\|____\| | | | | |  |
|  |  |  |  | ROTA1 | | | 01 | | 02 | \|____\|____\|____\| | | | | |  |
|  |  |  |  | ROTA2 | | | 01 | | 02 | \|____\|____\|____\| | | | | |  |
|  |  |  |  | PCV1 | | | 01 | | 02 | \|____\|____\|____\| | | | | |  |
|  |  |  |  | PCV2 | | | 01 | | 02 | \|____\|____\|____\| | | | | |  |
|  |  |  |  | PCV3 | | | 01 | | 02 | \|____\|____\|____\| | | | | |  |
|  |  |  |  | MEASLES | | | 01 | | 02 | \|____\|____\|____\| | | | | |  |
|  |  |  |  | VIT. A | | | 01 | | 02 | \|____\|____\|____\| | | | | |  |
|  |  |  |  | DEWORMING | | | 01 | | 02 | \|____\|____\|____\| | | | | |  |
|  | | Are there any vaccines given to the child (NAME) but not recorded on the card? | | 01 = Polio  02 = Measles  03 = Meningitis  04 = Hepatitis  88 = Others (specify) __________________  98= No, there is no  99 = Yes there is, but don’t know | | | | | | | | | | |  |
|  | | Do you have any certificate to a child (NAME) for completion of vaccination?  Check the certificate | | 01 = Yes (Certificate is presented)  02 = Yes (Certificate is not presented)  03 = No | | | | | | | | | | |  |
|  | | Has the child (NAME) had diarrhea in the last 2 weeks? | | 01 = Yes  02 = No | | | | | | | | | | | **2🡪529** |
|  | | Is there any blood in the stools? | | 01 = Yes  02 = No | | | | | | | | | | |  |
|  | | Is the child (NAME) still having diarrhea? | | 01 = Yes  02 = No | | | | | | | | | | |  |
|  | | During diarrhea, as compared to the usual how much fluid was offered to the child (NAME) to drink?  **(read the responses)** | | 01 = Much Less  02 = Somewhat less  03 = About the same  04 = More than usual  05 = Nothing to drink  98 = Did not start fluid  99 = Don’t Know | | | | | | | | | | |  |
|  | | During diarrhea, as compared to the usual how much food was offered to the child NAME) to eat?  **(read the responses)** | | 01 = Much Less  02 = Somewhat less  03 = About the same  04 = More than usual  05 = Never gave food  98 = Doesn’t start taking food  99 = Don’t Know | | | | | | | | | | |  |
|  | | For whom /non-family member/ you told about first that your child has diarrhea? | | 01 = HEW  02 = FLW  03 = Nurse  04 = TBA  05 = VCHW(HDA)  06 = Relative/friend/Neighbor  07 = Traditional healers  08 = Religious leader  09 = No one outside family  88 = Others (specify) __________________ | | | | | | | | | | |  |
|  | | Did you try to seek advice or treatment about child (NAME) diarrhea outside home? | | 01 = Yes  02 = No | | | | | | | | | | | **01**🡪**520** |
|  | | Even If you didn’t try to get advice about child (NAME) diarrhea from outside, did you give any treatment in your home? | | 01 = Yes  02 = No | | | | | | | | | | |  |
|  | | If your answer is **Yes Q. 517,** what did you do for child (NAME) diarrhea?  *Do not read responses*  *ASK: Anything else?*  *Record all responses* | | 01 = Continue to give breast milk  02 = Give fluid more than usual  03 = Continue to give food  04 = ORS  05 = Salt-sugar-fluid  06 = Child gets a rest  07 = Herbs/parsley/potion  08 = Medicine  88 = Others (specify) __________________ | | | | | | | | | | |  |
|  | | What were the reasons you did not seek treatment for the diarrhea?  *Do not read responses*  *ASK: Anything else?*  *Record all responses*  **Ask Only if the response Q 516 = 2** | | 01 = Not knowing where to go  02 = It is not serious illness  03 = It costs too much  04 = Health facility not open  05 = Transportation problem  06 = Not wanting to go alone  07 = Facility/poor quality service  08 = Not customary  09 = Due to household chore  10 = Husband didn’t allow  88 = Other (specify)_______________ | | | | | | | | | | |  |
|  | | If you sought advice or treatment from a health personnel, why did you seek the service?  *Do not read responses*  *ASK: Anything else?*  *Record all responses* | | 01 = Child has no improvement  02 = I have tried self-treatment but no improvement  03 = I have tried tradition medicine but not improved  04 = The disease became more severe  05 = I came for screening/growth monitoring  06 = I know the health care providers treat diarrhea  07 = Someone suggested me to take to health facility  08 = I came for immunization  88 = Other (specify) ____________________ | | | | | | | | | | |  |
|  | | If you sought advice or treatment from a health personnel or traditional practitioner, where (what place) did you seek the service?  *Do not read responses*  *ASK: Anything else?*  *Record all responses* | | 01 = Health Post  02 = Health Center  03 = Government Hospital  04 = Non-Governmental clinic  05 = Private Hospital  06 = Private clinic  07 = Private pharmacy  08 = Community based outlets  09 = Rural drug vendor  10 = Traditional practitioner  11 = holy water/ holy place  12 = TBA at home  88 = Other (specify) ____________________ | | | | | | | | | | | If >2 codes are encircled, go to next question  If only one code circled 🡪**523** |
|  | | Where did you first seek advice or treatment for child (Name) diarrhea?  *Use Number codes from question* ***521*** | | **________________________** | | | | | | | | | | |  |
|  | | How many days after the diarrhea began did you first seek advice or treatment for the child (NAME)?  *IF THE SAME DAY, RECORD '00'.* | | [ __________] Days after | | | | | | | | | | |  |
|  | | Can you tell us whether or not you were satisfied with the care given for diarrhea?  **(read all list of options)** | | 01 = Full satisfied  02 = Partly satisfied  03 = Neither satisfied nor dissatisfied  04 = Partly dissatisfied  05 = Dissatisfied  06 = I didn’t go to health post | | | | | | | | | | |  |
|  | | Was child (NAME) given any of the following to drink at any time since s/he started having the diarrhea: | | **Yes** | | | **No** | | | **Don’t know** | | | | |  |
|  |  | A fluid made from ORS sachet? | | 01 | | | 02 | | | 03 | | | | |  |
|  |  | Salt-sugar-fluid | | 01 | | | 02 | | | 03 | | | | |  |
|  |  | Other homemade fluid? | | 01 | | | 02 | | | 03 | | | | |  |
|  | | Was s/he given any modern medicine to treat diarrhea? | | 01 = Yes  02 = No  99 = Do not know | | | | | | | | | | |  |
|  | | What (else) was given to treat diarrhea?  *Do not read responses*  *ASK: Anything else?*  *Record all responses* | | **PILL OR SYRUP**  01 =Antibiotic  02 = Antihelminth  03 =Antimotility  04 = Zinc  05 = unknown pill or syrup  06 = other tablet/syrup (specify) ___________  **INJECTION**  07= Antibiotic  08 = Non-antibiotic  09 = Unknown injection.  88 = Other, specify___________________ | | | | | | | | | | |  |
|  | | Was anything (else) given to treat diarrhea rather than given medicine? | | 01 = Yes  02 = No  99 = Do not know | | | | | | | | | | |  |
|  | | Has a child (NAME) had an illness with a cough at any time in the last 2 weeks? | | 1 = Yes  2 = No | | | | | | | | | | | **02**🡪**549** |
|  | | When a child (NAME) had cough, did he/she breath faster than usual with short, rapid breaths or have difficulty breathing? | | 01 = Yes  02 = No | | | | | | | | | | | **02**🡪**532** |
|  | | When a child (NAME) had this illness, did the fast or difficult breathing happen due to a problem in the chest or due to a blocked or runny nose? | | 01 = Chest only  02 = Nose only  03 = Both  88 = Other, specify_______________  99 = Do not know | | | | | | | | | | |  |
|  | | When a child (NAME) had cough, did he/she have chest in-drawing? | | 01 = Yes  02 = No  99 = Do not know | | | | | | | | | | |  |
|  | | Is the child (NAME) still sick with a cough? | | 01 = Yes  02 = No | | | | | | | | | | |  |
|  | | How much was a child (NAME) offered to drink during the cough/breathing difficulty? | | 01 = Much Less  02 = Somewhat less  03 = About the same  04 = More than usual  05 = Nothing to drink  98 = Did not start fluid  99 = Don’t Know | | | | | | | | | | |  |
|  | | How much was a child (NAME) offered to eat during the cough/breathing difficulty? | | 01 = Much Less  02 = Somewhat less  03 = About the same  04 = More than usual  05 = Never gave food  98 = Doesn’t start taking food  99 = Don’t Know | | | | | | | | | | |  |
|  | | Who was the first non-family member you told that your child has coughed? | | 01 = HEW  02 = FLW  03 = Nurse  04 = TBA  05 = VCHW(HDA)  06 = Relative/friend/Neighbor  07 = Traditional healers  08 = Religious leader  09 = No one outside family  88 = Others (specify) __________________ | | | | | | | | | | |  |
|  | | Did you seek any advice or treatment for the cough outside home? | | 01 = Yes  02 = No | | | | | | | | | | | **01**🡪**541** |
|  | | If you didn’t try to seek any advice or treatment for the cough outside home, did you give any treatment to the child (NAME) in your house? | | 01 = Yes  02 = No | | | | | | | | | | |  |
|  | | If your answer is **Yes Q. 538,** what did you do for child (NAME) diarrhea?  *Do not read responses, ASK: Anything else?*  *Record all responses* | | 01 = Continue to give breast milk  02 = Give fluid more than usual  03 = Continue to give food  04 = Child gets a rest  88 = Others (specify) __________________ | | | | | | | | | | |  |
|  | | What were the reasons you did not seek for medical treatment?  **(Read out loud the responses)**  **Ask only if the response Q 537= 2** | | 01 = Not knowing where to go  02 = It is not serious illness  03 = It costs too much  04 = Health facility not open  05 = Transportation problem  06 = Not wanting to go alone  07 = Facility/poor quality service  08 = Not customary  09 = Due to household chore  10 = Husband didn’t allow  88 = Other (specify)_______________ | | | | | | | | | | |  |
|  | | If you sought advice or treatment from health personnel for child cough, why did you seek the service?  *Do not read responses*  *ASK: Anything else?*  *Record all responses* | | 01 = Child has no improvement  02 = I have tried self-treatment but no improvement  03 = I have tried tradition medicine but not improved  04 = The disease became more severe  05 = I came for screening/growth monitoring  06 = I know the health care providers treat diarrhea  07 = Someone suggested me to take to health facility  08 = I came for immunization  88 = Other (specify) ____________________ | | | | | | | | | | |  |
|  | | If you sought advice or treatment from a health personnel or traditional practitioner, From where (place) did you seek the service?  *Do not read responses ASK: Anything else?*  *Record all responses* | | 01 = Health Post  02 = Health Center  03 = Government Hospital  04 = Non-Governmental clinic  05 = Private Hospital  06 = Private clinic  07 = Private pharmacy  08 = Community based outlets  09 = Rural drug vendor  10 = Traditional practitioner  11 = holy water/ holy place  12 = TBA at home  88 = Other (specify) ____________________ | | | | | | | | | | | If >2 codes are encircled, go to next question  If only one code circled 🡪**544** |
|  | | Where did you first seek advice for child cough treatment?  Use Number codes from question.**542** | | ________________________ | | | | | | | | | | |  |
|  | | How many days after the cough began did you first seek advice or treatment for a child (NAME)?  *IF THE SAME DAY, RECORD '00'.* | | [ ____________-] Days later | | | | | | | | | | |  |
|  | | Can you tell us whether or not you were satisfied with the care given for cough?  Do not read list of options | | 01 = Full satisfied  02 = Partly satisfied  03 = Neither satisfied nor dissatisfied  04 = Partly dissatisfied  05 = Dissatisfied  06 = I didn’t go to health post | | | | | | | | | | |  |
|  | | At any time during the illness, did the child (NAME) take any drugs for the Illness (cough)? | | 01= Yes  02 = No  99 = I don’t know | | | | | | | | | | | **02🡪548**  **99🡪568** |
|  | | What drugs did the child(Name) take to treat cough?  Any other drug?  Record All Mentioned  Ask to see the packets of the drugs s/he gave the child. But if s/he doesn’t have any sample left, the interviewer has to show  Interviewer should have samples to help respondents identify the drug | | **ANT-BIOTIC**  01 = Bactrim  02 = Ampicillin  03 = Amoxicillin  04 = Chloramphenicol  05 = Tetracycline  06 = Other anti-biotic  **OTHER DRUGS**  07 = Aspirin  08 = Ibuprofen  09 = Paracetamol  88 = Other, specify_______________ | | | | | | | | | | |  |
|  | | Was anything given to treat cough rather than given medicine? | | 01 = Yes  02 = No  99 = Do not know | | | | | | | | | | |  |
|  | | Has child (NAME) been ill with a fever at any time in the last 2 weeks? | | 01= Yes  02 = No | | | | | | | | | | | **2🡪566** |
|  | | Has child (NAME) been ill with other disease besides the fever at any time in the last 2 weeks? | | 01 = Malaria  02 = Cough  03 = Ear pain  04 = Measles  05 = No other illness  88 = others (specify) __________________  99 = I don’t know | | | | | | | | | | |  |
|  | | Is the child (NAME) still sick with a (fever)? | | 01= Yes  02 = No | | | | | | | | | | |  |
|  | | How much was a child (NAME) offered to drink during the fever? | | 01 = Much Less  02 = Somewhat less  03 = About the same  04 = More than usual  05 = Nothing to drink  98 = Did not start fluid  99 = Don’t Know | | | | | | | | | | |  |
|  | | How much was the child (NAME) offered to eat during the fever? | | 01 = Much Less  02 = Somewhat less  03 = About the same  04 = More than usual  05 = Never gave food  98 = Doesn’t start taking food  99 = Don’t Know | | | | | | | | | | |  |
|  | | Who was the first non-family member you told that your child has fever? | | 01 = HEW  02 = FLW  03 = Nurse  04 = TBA  05 = VCHW(HDA)  06 = Relative/friend/Neighbor  07 = Traditional healers  08 = Religious leader  09 = No one outside family  88 = Others (specify) __________________ | | | | | | | | | | |  |
|  | | Did you seek any advice or treatment for child (NAME) fever outside home? | | 01 = Yes  02 = No | | | | | | | | | | | **01**🡪**558** |
|  | | What did you do for the child (NAME) in your home during the fever?  *You can get more than one response so read all and*  *ASK: Anything else?* | | 01 = Continue to give breast milk  02 = Give fluid more than usual  03 = Continue to give food  04 = Child gets a rest  05 = cooling the body  88 = Others (specify) __________________ | | | | | | | | | | |  |
|  | | What were the reasons you did not seek medical treatment for child (NAME) during the fever?  *You can get more than one response so read all and*  *ASK: Anything else?* | | 01 = Not knowing where to go  02 = It is not serious illness  03 = It costs too much  04 = Health facility not open  05 = Transportation problem  06 = Not wanting to go alone  07 = Facility/poor quality service  08 = Not customary  09 = Due to household chore  88 = Other (specify)_______________ | | | | | | | | | | |  |
|  | | If you sought advice or treatment from health personnel for child’s fever, why did you seek the service?  *Do not read responses*  *ASK: Anything else?*  *Record all responses* | | 01 = Child has no improvement  02 = I have tried self-treatment but no improvement  03 = I have tried tradition medicine but not improved  04 = The disease became more severe  05 = I came for screening/growth monitoring  06 = I know the health care providers treat diarrhea  07 = Someone suggested me to take to health facility  08 = I came for immunization  88 = Other (specify) ____________________ | | | | | | | | | | |  |
|  | | If you sought advice or treatment from a health personnel or traditional practitioner, where (what place) did you seek the service?  *Do not read responses*  *ASK: Anything else?*  *Record all responses* | | 01 = Health Post  02 = Health Center  03 = Government Hospital  04 = Non-Governmental clinic  05 = Private Hospital  06 = Private clinic  07 = Private pharmacy  08 = Community based outlets  09 = Rural drug vendor  10 = Traditional practitioner  11 = holy water/ holy place  12 = TBA at home  88 = Other (specify) ____________________ | | | | | | | | | | | If >2 codes are encircled, go to next question  If only one code circled 🡪**561** |
|  | | Where did you go first to seek advice to treat child (NAME) fever?  Use Number codes from question **559** | | ________________ | | | | | | | | | | |  |
|  | | How many days after the (fever) began did you first seek advice or treatment for (NAME)?  *IF THE SAME DAY, RECORD '00'.* | | [ _________________] Days later | | | | | | | | | | |  |
|  | | Can you tell us whether or not you were satisfied with the care given for cough?  Do not read list of options | | 01 = Full satisfied  02 = Partly satisfied  03 = Neither satisfied nor dissatisfied  04 = Partly dissatisfied  05 = Dissatisfied  06 = I didn’t go to health post | | | | | | | | | | |  |
|  | | At any time during the illness, did the child (NAME) take any drugs for the Illness (fever)? | | 1= Yes  2 = No  99 = I don’t know | | | | | | | | | | | **02🡪566**  **99**🡪**566** |
|  | | What drugs did the child(Name) take?  Any other drug?  Record All Mentioned  If the respondent has given drug for the child but doesn’t know the name of the drug, ask to see the packets of the drugs s/he gave the child. But if s/he doesn’t have any sample left, the interviewer has to show  Interviewer should have samples to help respondents identify the drug | | **ANTIMALARIAL DRUGS**  01 = Fansidar/sp  02 = Chloroquine  03 = COARTEM  04 = Quinine  05 = Other anti- malarial  **ANTIBIOTIC**  06= Bactrim  07 = Ampicillin  08 = Amoxicillin  09 = Chloramphenicol  10 = Tetracycline  11 = Other anti-biotic  **OTHER DRUGS**  12 = Aspirin  13 = Ibuprofen  14 = Paracetamol  88 = Other, specify_______________ | | | | | | | | | | |  |
|  | | Have you given other than modern medicine to treat the fever> | | 01= Yes, specify __________________  02 = No  99 = Do not know | | | | | | | | | | |  |
|  | | Does your household have any functional bed nets that can be used while sleeping? | | 01= Yes  02 = No | | | | | | | | | | | **02🡪600** |
|  | | How many functional mosquito nets does your household have? | | Number of nets_______________ | | | | | | | | | | |  |
|  | | Did anyone in the family sleep under mosquito net(s) last night? | | 1= Yes  2 = No  99 = Do not know | | | | | | | | | | | **02🡪600**  **99🡪600** |
|  | | **If yes Q. 568**, who slept under mosquito net(s) last night?  (multiple answer)  Probe: anyone else? | | 01 = The child who is eligible for this study?  02 = Self/the person who answering these questions  03 = Newborn baby  04 = Other children (under 5)  05 = Head of the household/Father  88 = Others (specify) _____________________ | | | | | | | | | | |  |
| **Section VI: Awareness and Perceived Quality of iCCM Services** | | | | | | | | | | | | | | | |
| **Interviewer:** *Now I want to talk to you about community health services* | | | | | | | | | | | | | | | |
|  | | Do you know that health post provides treatment service to children under the age of five years? | | 01= Yes  02 = No | | | | | | | | | | | **2🡪603** |
|  | | Tell me the kind of illnesses/disease you know they usually treat? | | 01 = Diarrhea  02 = Cough  03 = Fever  04 = Skin rash  05 = Weight loss  06 = Ear infection  07 = Measles  88 = Other__________________________ | | | | | | | | | | |  |
|  | | From whom did you learn that they provide the medical/treatment services at the health post? You can circle more than one. (Multiple response is possible)  Do not read responses | | 01 = Kebele meeting  02 = HDA visiting  03 = HEWs home to home visit  04 = From neighbors, family member/relatives  05 = When visiting Health Post for other services  06 = Women association meeting  07 = Religious leaders/meetings  88 = Others___________________________ | | | | | | | | | | |  |
|  | | Have you visited a health post in the last one year to get treatment for your child? | | 1= Yes  2 = No | | | | | | | | | | | **02 🡪** end of interview |
|  | | Where did you go first to get treatment for your sick child? | | 01 = Health Post  02 = Health Center  03 = Government Hospital  04 = Other Places (Specify) ________________ | | | | | | | | | | |  |
|  | | How many times did you visit the health post/health center to get treatment for your sick child in the last one year? | | 01 = One time  02 = Two times  03 = Three times  04 = Four or more times | | | | | | | | | | |  |
|  | | Recently/during your last visit, what was the illness that made you take your child for treatment? | | 01 = Diarrhea  02 = Cough  03 = Fever  04 = Skin rash  05 = Weight loss/malnutrition  06 = Ear infection  88 = Other__________________________ | | | | | | | | | | |  |
|  | | In general, how could you rate the service you received from the facility? | | 01 = Full satisfied  02 = Partly satisfied  03 = Neither satisfied nor dissatisfied  04 = Partly dissatisfied  05 = Dissatisfied | | | | | | | | | | |  |
|  | | If you visited Health post/health center for the child who was sick in the last two weeks, were you referred to other health facility, or get treated at the HP? | | 01 = referred to higher health facility  02 = Treated at Health post  03 = Did not get treatment | | | | | | | | | | |  |
|  | | If you visited Health post/ health center for the child who was sick in the last two weeks, Did the health care provider tell you to return to the health facility if you see any of the danger signs? | | 01 = Yes  02 = No  03 = Didn’t go to health facility | | | | | | | | | | |  |
|  | | Did the provider tell you about any signs or symptoms you may see for which you must immediately bring the child back?  IF YES, can you tell me what these are ?Probe if necessary | | 01 = Fever  02 = Breathing Problem  03 = Become sicker  04 = Blood in stool Vomiting  05 = Poor/Not Eating  06 = Poor/Not Drinking  88 = Other________________________  98 = No, None  99 = DON’T KNOW | | | | | | | | | | |  |
|  | | Did the service provider give or prescribe any medicines for the child [NAME] to take at Home? | | 01 = Yes  02 = No | | | | | | | | | | | **02 🡪 613** |
|  | | Did the service provider at the facility explain to you how to give these medicines to the child [NAME] at home? | | 01 = Yes  02 = No | | | | | | | | | | |  |
|  | | Did the service provider at the facility explain to you how to feed the child [NAME] at home? | | 01 = Yes  02 = No | | | | | | | | | | | **02 🡪 615** |
|  | | What did the service provider counseling you about child feeding, giving fluid (or breast milk, if the child [Name] is ill? | | 01 = Continue breast feeding  02 = Breast feeding more than the usual  03 = Continue to give food  04 = Give food more than the usual  05 = Continue giving fluid to drink  06 = Give fluid more than the usual  88 = Other (Specify) ______________________ | | | | | | | | | | |  |
|  | | Can you rate your level of satisfaction in terms of the list of indicators of service satisfaction? | | **FS** | | **PS** | | **NS/NDS** | | **PDS** | **DS** | | | |  |
|  |  | Waiting time for service | | 01 | | 02 | | 03 | | 04 | 05 | | | |  |
|  |  | Skill of personnel provided care and treatment | | 01 | | 02 | | 03 | | 04 | 05 | | | |  |
|  |  | Completeness of equipment and supplies | | 01 | | 02 | | 03 | | 04 | 05 | | | |  |
|  |  | Ability of the HEW/FLW to discuss your child’s problem or health issue | | 01 | | 02 | | 03 | | 04 | 05 | | | |  |
|  |  | Quality of examinations  and treatment provided | | 01 | | 02 | | 03 | | 04 | 05 | | | |  |
|  |  | Availability of medicine | | 01 | | 02 | | 03 | | 04 | 05 | | | |  |
|  |  | The working hours of health facility | | 01 | | 02 | | 03 | | 04 | 05 | | | |  |
|  |  | Cleanliness of the facility | | 01 | | 02 | | 03 | | 04 | 05 | | | |  |
|  |  | The cost of the visit and treatment provided | | 01 | | 02 | | 03 | | 04 | 05 | | | |  |
|  |  | The privacy that was provided at the facility | | 01 | | 02 | | 03 | | 04 | 05 | | | |  |
|  | | In general how could you rate the service you received from the facility? | | 01 | | 02 | | 03 | | 04 | 05 | | | |  |

***FS = Fully Satisfied; PS = Partially satisfied; NS/NDS= never satisfied nor dissatisfied; PDS = Partially dis-satisfied; DS = Dis-satisfied***

**That is the end of our interview. Thank you very much for taking the time to answer these questions.**

Time at end of interview: ____:____

**THANK YOU!!**
