# Supplementary material for: Changes in care-seeking for common childhood illnesses in the context of Integrated Community Case Management (iCCM) program implementation in Benishangul Gumuz region of Ethiopia
Source: PLoS One. 2020 Nov 13;15(11):e0242451. doi: 10.1371/journal.pone.0242451 (PMC7665800; doi:10.1371/journal.pone.0242451)
Supplement: Attachment — Submitted filename: Point by point PLOS.docx [file pone.0242451.s004.docx]

Comment from the editor: English language correction is deemed necessary.

The manuscript is now edited further.

Comments of Reviewer 1

Comment 1: The authors have not fully made all data underlying the findings.

Response: We disagree with the evaluation of the reviewer. We have already made our data available as a supporting table (S2 table).

Comment 2: Previous Comment 1: The authors have explained the meaning of ‘emerging’ but the ‘word’ does not make sense in the title.

Response: The title is now modified accordingly.

Comment 3: Previous Comment 2: Unsure if Ethiopia system does not need the observational studies to be registered. For most registries all types of research need to be registered.

Response: As the reviewer mentioned, observational studies can also be registered but we don’t think that’s mandatory. That’s why the study was not registered.

Comment 4: Previous Comment 5: Description of the intervention has been added still unclear what support other than medicines and improving linkages was done by research team It is unclear if was supervision was by the health system.

Response: Please note that this is an observational study so that the researchers were not directly involved in the implementation of the program. But if the concern of the reviewer is on the role Emory University in the implementation of iCCM program, the following activities were implemented by Emory University: training of frontline health workers, consolidating referral linkage between health posts and health centers, instating quality improvement framework at various levels of the system, provision of supervisory skill training for health extension worker supervisors, joint supportive supervision and monthly couching of health extension workers. We have now made some correction on the section “Description of the intervention” to make the issue clear for readers.

Comment 5: Previous Comment 7: Has this been added as the limitation of the trial.

Response: this is now listed as the limitation in the last paragraph of the discussion section (Page 24)

Comment 6: Previous Comment 8: Is there a possibly if the same child could have been selected in all 3 surveys. If yes, would that primed the caregivers with the responses to be given.

Response: It would be very unlikely for a child to be included in multiple surveys for two reasons: (1) the chance a child would be sick during two or more survey rounds is obviously low, (2) in each survey round a random and independent sample of children were selected. So we don’t think the concern of the reviewer is a major concern in our study

Comment 7: Previous Comment 11: Trial profile is still missing.

Response: It would be more interesting to have a flow chart that describe the characteristics of the study subjects excluded from the study, unfortunately we did not collect data on subjects excluded from the study.

Comment 8: Previous Comment 13: Not added in limitation as mentioned by author.

Response: We disagree with the evaluation reviewer. As you may read from the last paragraph of the discussion section, we have discussed the issue as a limitation “Furthermore, infants 2-11 months were underrepresented (contributed to only 5-6% of the total children enrolled) which may limit the generalizability of the findings of the study.”

Comment 9: Previous Comment 14: Should add as a limitation.

Response: We have now stated this as a limitation in the last sentence of the discussion section (Page 24).
